# Maternal and Neonatal Vitamin D Binding Protein Polymorphisms and 25-Hydroxyvitamin D Cutoffs as Determinants of Neonatal Birth Anthropometry

**DOI:** 10.3390/nu14183799

**Published:** 2022-09-15

**Authors:** Spyridon N. Karras, Erdinç Dursun, Merve Alaylıoğlu, Duygu Gezen-Ak, Stefan Pilz, Cedric Annweiler, Fatme Al Anouti

**Affiliations:** 1 Laboratory of Biological Chemistry, Medical School, Aristotle University, 55535 Thessaloniki, Greece; 2 Brain and Neurodegenerative Disorders Research Laboratories, Department of Neuroscience, Institute of Neurological Sciences, Istanbul University-Cerrahpasa, 34320 Istanbul, Turkey; 3 Division of Endocrinology and Diabetology, Department of Internal Medicine, Medical University of Graz, 8036 Graz, Austria; 4 Department of Geriatric Medicine and Memory Clinic, Research Centre on Autonomy and Longevity, University Hospital, 49933 Angers, France; 5 Robarts Research Institute, Department of Medical Biophysics, Schulich School of Medicine and Dentistry, The University of Western Ontario, London, ON N6A 3K7, Canada; 6 Department of Health Sciences, College of Natural and Health Sciences, Zayed University, Abu Dhabi P.O. Box 144534, United Arab Emirates

**Keywords:** vitamin D, pregnancy, polymorphism, birth anthropometry

## Abstract

Background: Vitamin D-binding protein (VDBP) is a vital regulator of optimal vitamin D homeostasis and bioavailability. Apart from its well-documented role as a key component in vitamin D dynamic transfer and circulation, it has a myriad of immunoregulatory functions related to innate immunity, which becomes particularly critical in states of increased immunological tolerance including pregnancy. In this regard, VDBP dyshomeostasis is considered to contribute to the development of several fetal, maternal, and neonatal adverse outcomes. However, precise physiological pathways, including the contribution of specific VDBP polymorphisms behind such phenomena, are yet to be fully deciphered. Our aim was to assess the combined effect of maternal and neonatal VDBP polymorphism heterogeneity in conjunction with different maternal and neonatal 25(OH)D cutoffs on the neonatal anthropometric profile at birth. Methods: The study included data and samples from a cohort of 66 mother–child pairs at birth. The inclusion criterion was full-term pregnancy (gestational weeks 37–42). Neonatal and maternal 25(OH)D cutoffs were included according to vitamin D status at birth and delivery. Concentrations of 25(OH)D2 and 25(OH)D3 were measured using liquid chromatography–tandem mass spectrometry. Results: The upper arm length of neonates with 25(OH)D ≤ 25 nmol/L was higher in neonate CC carriers for rs2298850. The upper thigh neonatal circumference was also higher in the ones with either 25(OH)D ≤ 50 or ≤75 nmol/L in rs2298850 CG + GG or rs4588 GT + TT carriers. We did not observe any significant effect for maternal VDBP polymorphisms nor for birth maternal 25(OH)D concentrations, on birth neonatal anthropometry. Conclusions: Our findings emphasize a potential role for neonatal VDBP genotypes rs2298850 and rs4588, in conjunction with specific neonatal 25(OH)D cutoffs, in the range of sufficiency on neonatal growth and development.

## 1. Introduction

Pregnancy is a distinctive dynamically active state, where several factors pertaining to maternal health and nutrition [1,2], as well as genetic determinants, comprise the primary regulators of fetal in utero, growth and development, and future offspring health in childhood and adult life [3,4]. Birth anthropometry is one of the well-established clinical parameters that reflect neonatal health status when assessed immediately after birth. It has also been considered a vital determinant of future offspring metabolic outcomes including obesity and cardiovascular diseases (CVD) [5].

The concept of association between birth anthropometry including birth weight and adult cardiovascular health was first pinpointed by the research of David Barker [6], who concluded that small for gestational age (SGA) infants had a higher mortality rate due to CVD compared to those with normal birth weight.

According to a widely adopted hypothesis, spurred by several observational findings, hypovitaminosis D during pregnancy has been correlated with an increased risk for the development of adverse pregnancy and offspring outcomes [7,8,9,10]. This hypothesis was mainly based on previous mechanistic evidence that revealed the strong association between maternal 25-hydroxy-vitamin D [25(OH)D] and serum fetal (cord blood) 25(OH)D concentrations [11,12]. On the other hand, available results on the relationship between the maternal and neonatal vitamin D profile with offspring birth anthropometry are conflicting. While several multiethnic prospective cohort studies have consistently reported that maternal vitamin D concentrations were inversely associated with neonatal birth weight, knee–heel length, and other neonatal anthropometric parameters [13,14,15,16,17,18], such results were not confirmed by other observational studies [19,20,21,22,23,24].

Studies pertaining to maternal vitamin D supplementation during pregnancy for the purpose of improving neonatal birth anthropometric profiles have failed to exhibit a consistent benefit [25,26]. It has become evident that additional ethnic variations in vitamin D—metabolism genes and specific polymorphisms [27]—as well as country-specific dietary patterns [28] and differences in ultraviolet B (UVB) exposure [29], could partly bridge this gap in scientific knowledge and explain the discrepancies reported across research studies. Our group has previously disclosed results from a maternal–neonatal pair cohort, which indicated that maternal TAQI vitamin D receptor (VDR) polymorphism significantly impacts neonatal birth anthropometry when maternal 25(OH)D concentrations are ≥50 nmol/L, whereas this amalgamation, depicts a marginal effect in the presence of a neonatal TAQI polymorphism with neonatal 25(OH)D values [27].

Vitamin D-binding protein (VDBP) also acts as one of the main regulators for determining vitamin D status by impacting its homeostasis [16]. VDBP is encoded by the GC gene, which is located on chromosome 4 (4 q11–13) [30,31,32,33,34]. Recent findings indicate that the disruption of the VDBP equilibrium comprises a risk factor for unfavorable fetal, maternal, and neonatal outcomes [35,36,37,38,39]. We have recently reported that mothers with the CC genotype for rs2298850 and rs4588 manifested higher 25(OH)D concentrations [40]. Former findings on the influence of specific maternal–neonatal VDBP polymorphisms on neonatal birth anthropometry remain limited. A previous study [41] revealed that GC SNPs rs12512631 and rs7041 significantly altered the interactions between maternal and cord-blood concentrations of 25(OH)D and birth weight, resulting in a reduction in the neonatal birth weight in the offspring of mothers possessing specific alleles and polymorphisms.

Our main hypothesis was that by adopting specific maternal and neonatal cutoffs for vitamin D status in conjunction with maternal–neonatal VDBP polymorphisms, specific effects on the neonatal anthropometry profile at birth would be identified. Consequently, in this study, we assessed the combined influence of maternal and neonatal VDBP polymorphisms and different maternal and neonatal 25(OH)D cutoffs on neonatal birth anthropometry.

## 2. Methods

### 2.1. Participants

This study utilized data from samples collected from a cohort of 70 mother–child pairs at birth as previously described [11]. All women were fair skinned. The inclusion criterion was full-term pregnancy (gestational weeks 37–42). Maternal exclusion criteria were primary hyperparathyroidism, secondary osteoporosis, heavy alcohol use (≥7 alcohol units per week or ≥6 units at any time during pregnancy), hyperthyroidism, nephritic syndrome, inflammatory bowel disease, rheumatoid arthritis, osteomalacia, obesity (body mass index (BMI) >30 kg/m^2^), gestational diabetes, and the use of any medications affecting calcium (Ca) or vitamin D status (e.g., corticosteroids), including vitamin D supplements. Neonatal exclusion criteria were being small-for-gestational age (SGA) and existence of serious congenital anomalies. All mothers provided consent for the study, which was conducted between January 2018 and September 2018. The study protocol was approved by Bioethics Committee of the Aristotle University of Thessaloniki, Greece (approval number 1/19-12-2011).

### 2.2. Biochemical and Hormonal Assays

Maternal blood samples were collected 30–60 min prior to delivery, while umbilical cord blood was obtained after clamping. Biochemical analysis for Ca, phosphorus (P), parathyroid hormone (PTH), 25-hydroxyvitamin D_2_ [25(OH)D_2_], and 25(OH)D was performed as previously described [11,42]. For 25(OH)D_2_ and 25(OH)D, concentrations were measured by liquid chromatography–tandem mass spectrometry (LC-MS/MS), with lower limits of quantification (LLOQ): 25(OH)D_2_ (0.5 ng/mL), 25(OH)D(0.5 ng/mL); total 25 (OH)D was determined by the sum of both forms [42].

### 2.3. Demographic and Anthropometric Data

Demographic and social characteristics of the participants were recorded upon enrollment. Umbilical cord blood samples collected at the time of delivery were stored in aliquots along with plasma and serum at −70 °C until assays were performed. We also evaluated neonatal anthropometry at birth. All neonatal anthropometric measurements and assessments were conducted by the same trained nurse, between 12 and 72 h of age according to standard techniques [43]. The following parameters were recorded: birth weight, height, neck–rump length, upper arm, femur, and knee–heel lengths; head, chest, abdominal, upper arm, and middle thigh circumferences; and anterior chest and abdominal skin fold thickness. Birth weight of the neonates was measured on regularly calibrated scales. Knee–heel length was measured with a hand-held BK5 infant knemometer (Force Technology, Brondby, Denmark). Instrument software calculated the mean of 10 sequential readings and generated a printed report of all readings and the calculated mean. Neonatal height was measured to the nearest millimeter using an Ellard newborn length board (Ellard Instrumentation Ltd., Seattle, WA, USA). Abdominal, upper arm and middle thigh head, mid-upper arm, and maximal head circumferences were measured using a plastic encircling tape (Child Growth Foundation, London, UK). Abdominal skin fold was measured using Holtain calipers (Holtain, Crymych, UK).

### 2.4. Neonatal and Maternal Vitamin D Status Cutoffs and Combined VDBP Polymorphisms Evaluation

Neonatal and maternal VDBP polymorphisms were utilized to determine differences in the vitamin D status patterns as per the following cutoffs at birth: 25(OH)D ≤ 25 nmol/L (deficiency), 25–50 nmol/L (insufficiency), and 25(OH)D ≥ 50 nmol/L (sufficiency) [44,45]. 

### 2.5. VDBP Analysis

DNA isolation, real-time PCR (RT-PCR), and genotyping of VDBP SNPs were all performed according to manufacturer’s protocol, as described previously [46].

### 2.6. UVB Measurements

UVB data were collected at the Laboratory of Atmospheric Physics, School of Physics, Aristotle University of Thessaloniki. The daily integral of vitamin D effective UVB radiation (09:00 to 16:00 local time) was represented by the amount of sunlight hitting a horizontal surface, in Watts per hour square meter (wh/m^2^). Mean UVB exposure over the past 45 days (daily integral) prior was determined.

### 2.7. Statistical Analysis

For statistical analysis, “SPSS 24.0” software(SPSS, Chicago, IL, USA) was used. Distributions of genotypes of VDBP polymorphisms were analyzed with Chi-square (χ^2^) test, df:2 for genotypes. Significance was confirmed with Cramer’s V/Kendall’s tau-c and comparisons between mean values of the groups were performed with One-way ANOVA followed by multiple comparison tests: either Tukey HSD or Dunnett C depending on the normality of the data set. Homogeneity of variances was checked with Levene’s Test. The data and *p*-values were adjusted if required for maternal and paternal height (cm), BMI pre-pregnancy (kg/m^2^), BMI terminal (kg/m^2^),UVB exposure, and weeks of gestation by One-way analysis of covariance(ANCOVA). All data are presented as the mean ± SD in the text and figure legends. *p*-values lower than 0.05 were considered statistically significant.

## 3. Results

Seventy mother–neonate pairs were initially recruited. Four neonates were excluded from the analysis because of missing data. The demographic and laboratory data of mothers and neonates are presented in Table 1.

### 3.1. Birth Neonatal Anthropometry According to Maternal Vitamin D Status at Birth and Maternal VDBP Polymorphisms

When neonatal anthropometry was assessed according to maternal VDBP polymorphisms using different maternal cutoffs for 25(OH)D at birth, our results revealed that height and upper arm length parameters of neonates were significantly higher in mothers with 25(OH)D ≤ 25 nmol/L and those harboring the CC genotype for rs2298850 (Table 2). When neonatal parameters were compared among mothers with 25(OH)D ≤ 50 nmol/L, neonatal weight and upper arm length were found to be higher in mothers with the CC genotype for rs2298850, while only upper arm length was higher in maternal CC genotype carriers for rs4588 (Table 3). However, all results were non-significant after adjusting for maternal height (cm), BMI pre-pregnancy (kg/m^2^), BMI at term (kg/m^2^), UVB exposure, and weeks of gestation. There was no statistical significance in birth neonatal anthropometry among mothers with 25(OH)D ≤ 75 nmol/L (Table 4).

### 3.2. Birth Neonatal Anthropometry According to Neonatal Vitamin D Status at Birth and Neonatal VDBPPolymorphisms

Our findings indicated that upper arm length, lower leg calf circle, and knee–heel length of neonates with 25(OH)D ≤25 nmol/L were higher in CC genotype carriers for rs2298850, while only upper arm length was higher in GG genotype for rs7041. Upon adjustment of the data according to maternal height (cm), BMI pre-pregnancy (kg/m^2^), BMI at term (kg/m^2^), UVB exposure, and weeks of gestation, only upper arm length remained statistically significant for rs2298850 (Table 5). Neonatal anthropometric parameters, including neck–rump length and high thigh circumference, were found to be higher in CG + GG carriers of rs2298850 and GT + TT carriers of rs7041, as well as neck–rump length, high thigh circumference, and knee–heel length, in CA + AA carriers for rs4588 in both neonates with 25(OH)D ≤50 nmol/L and <75 nmol/L. Nevertheless, only high thigh circumference remained significant for rs2298850 and rs4588 after adjustments (Table 5, Table 6 and Table 7).

## 4. Discussion

We aimed to evaluate the effects of maternal and neonatal VDBP polymorphisms on neonatal anthropometry at birth, according to several cutoffs of vitamin D status. 

Results from this maternal–neonatal pair cohort indicated that:

(i) Upper arm length of neonates with 25(OH)D <25 nmol/L was higher in CC carriers for rs2298850.
(ii) Upper thigh circumference of neonates was also higher in the ones with either 25(OH)D ≤50 or ≤75 nmol/L, in rs2298850 CG+GG, or in rs4588 GT+TT carriers.
(iii) We did not observe any significant confounder effect either for maternal VDBP polymorphisms or for birth maternal 25(OH)D concentrations on birth neonatal anthropometry. These findings emphasize that neonatal genetic background in conjunction with attained 25(OH)D concentrations at birth could result in different anthropometric patterns at birth. We have previously highlighted the significance of population-specific genetic profiling in providing a more thorough view of vitamin D deficiency among neonates and their mothers [27]. However, there was a lack of concurrent evaluations of both maternal and neonatal VDBP polymorphisms in the context of different 25(OH)D thresholds oriented towards a detailed assessment of birth anthropometry as a method of crude estimation of neonatal adiposity, which could ascertain an adverse metabolic offspring profile in adult life.

Although VDBP polymorphisms have been reported to influence 25(OH)D concentrations during pregnancy in different populations, results remain largely heterogeneous. In specific, rs12512631 and rs7041 were found to affect maternal and cord-blood concentrations of 25(OH)D [47,48]. Genetic variations in VDBP polymorphisms could also partly justify differences in response to vitamin D supplementation during pregnancy since the GC rs2282679 polymorphism was associated with achieved 25(OH)D concentrations after cholecalciferol supplementation [49].The influence of the genetic variants of rs17467825, rs4588, rs2282679, andrs2298850 on maternal 25(OH)D has been reported to be altered by vitamin D supplementation and sunshine exposure [50]. These results highlight that specific vitamin D polymorphisms could influence maternal and neonatal vitamin D status; however, available data on the relationship between maternal 25(OH)D concentrations during pregnancy and neonatal anthropometry are still conflicting.

Two meta-analyses on observational and supplementation studies previously published in the field revealed a modest proof regarding the association between maternal 25(OH)D status and offspring birthweight [51,52]. Data from three observational studies confirmed the existence of a limited positive relationship between maternal 25(OH)D and offspring birthweight, using log-transformed 25(OH)D concentrations after adjustment for confounding factors (pooled regression coefficient 5.63 g/10% changes maternal 25(OH)D, 95% confidence interval (CI) 1.11 to 10.16 g), but results were not evident across intervention-based investigations [51]. Another review of twenty-four clinical trials with a total of 5405 participants summarized the impact of vitamin D supplementation during pregnancy on offspring growth, morbidity, and mortality. The examined data revealed that neonates whose mothers received vitamin D supplementation during pregnancy had a higher weight at birth ((mean difference [MD], 75.38 g; 95% CI, 22.88 to 127.88 g), 3 months (MD, 0.21 kg; 95% CI, 0.13 to 0.28 kg), 6 months (MD, 0.46 kg; 95% CI, 0.33 to 0.58 kg), 9 months (MD, 0.50 kg; 95% CI, 0.01 to 0.99 kg), and 12 months (MD, 0.32 kg; 95% CI, 0.12 to 0.52 kg)) [52]. Recent results also demonstrated that maternal 25(OH)D concentration ≤ 20 ng/mL correlated with lower birthweight (−308 g; −540, −76) and lower ponderal index [53]. Higher maternal BMI (≥25 kg/m^2^) in conjunction with 25(OH)D ≤50 nmol/L at 10–14 gestational weeks (GW) was associated with a lower birthweight z-score (0.56; 95% CI: −0.99, −0.13) and length (−1.56 cm; 95% CI: −3.07, −0.06), while at 23–31 GW it was associated with shorter length (−2.77 cm; 95% CI: −13.38, −4.98) and a lower sum of skin folds (−9.18 mm; 95% CI: −13.38, −4.98). Results were strikingly different for women with normal BMI and 25(OH)D <50 nmol/L (larger birthweight z-scores (0.64; 95% CI: 0.03, 1.25) and at 33–39 GW with both higher birthweight z-score (1.22; 95% CI: 0.71, 1.73), and longer length (1.94 cm; 95% CI: 0.37, 3.52)) [53]. Higher maternal total and free 25(OH)D concentrations were similarly linked to higher neonatal birthweight in a retrospective maternal–neonatal Australian cohort following adjustment for confounders and covariates including maternal age, BMI, and ethnicity [54].

However, few other researchers have reported a lack of association between maternal 25(OH)D concentrations with newborn anthropometry or cord-plasma variables. These neutral results were demonstrated among European, Indian, and other multiethnic cohorts [55,56]. Moreover, no association was observed between maternal–neonatal concentrations of vitamin D and weight and height values in two and four-year offspring [57]. Despite the existence of gaps in the current research literature and a profound diversity in the research design, most studies indicate a positive mechanistic association between maternal–neonatal vitamin D equilibrium and neonatal birth anthropometry. The link, however, between VDBP polymorphisms, vitamin D status, and offspring anthropometric profiles was unclear, mostly due to the scarcity of available studies.

A previous Korean study [41] reported that GC SNPs rs12512631 and rs7041 significantly modified the relationships between maternal and cord-blood concentrations of 25(OH)D and birth weight. In detail, GC SNPs rs12512631 and rs7041altered the interaction between the maternal and cord-blood concentrations of 25(OH)D and birth weight significantly, hence providing implications for a functional interaction of maternal vitamin D status and VDBP polymorphisms with decreased birth weight. Researchers further revealed that hypovitaminosis D among neonates was significantly correlated with reduced birth weight only among infants whose mothers were carriers of the rs7041 ‘G’ allele. However, this analysis focused only on birth weight as an anthropometric outcome and did not include the various cutoffs of maternal vitamin D status and neonatal VDBP polymorphisms. In our analysis, we included several anthropometric neonatal parameters, as well as evaluated neonatal VBDP polymorphisms and UVB measurements, to control for seasonal variations in vitamin D status.

### Strengths and Limitations

The strength of our study lies in its comprehensiveness in terms of interpreting genotyping data in conjunction with serum 25(OH)D cutoffs for both mothers and neonates on offspring body anthropometry, while accounting for additional determinants of vitamin D status including diet, UVB exposure, and supplement use. This research could inform future studies aiming to better understand the role of functional polymorphisms for maternal VDBP genetic variants in conjunction with a neonatal cutoff, which is largely dependent on maternal stores, pregnancy complications, and neonatal growth and development. We acknowledge that the investigation has a few limitations. First, the sample size was small and not powered to detect additional differences in other maternal–neonatal cutoffs. Nonetheless, it was sufficiently powered to show significant differences regarding the main aim of the study. Second, the cross-sectional design of the study could not prove a causal relationship. Third, all the women were Caucasian, so our results cannot be safely generalized to other ethnicities known to differ at least in the frequency of VDBP polymorphisms, indicating that further similar studies from other regions could be useful in order to elucidate the full extent of the ethnic VDBP variation effect in neonatal outcomes. On the other hand, the inclusion of both maternal and neonatal polymorphisms as well as the assessment of different cutoffs could provide a realistic overview of maternal–neonatal dynamics, which is absent in most previous studies of a similar design.

## 5. Conclusions

In conclusion, these findings emphasize a potential role for neonatal VDBP genotypes rs2298850 and rs4588, in conjunction with specific neonatal 25(OH)D cutoffs, in the range of sufficiency on neonatal growth and development. Further and larger studies are required to elucidate the exact dynamic pathways of maternal VDBP and genetic variants on pregnancy complications and offspring birth anthropometry.

## Figures and Tables

**Table 1:** Maternal and neonatal demographic and anthropometric characteristics.

Maternal	
Number (*n*)	66
Age (years)	31.92 ± 6.08
Height (cm)	164.85 ± 5.47
Weight; pre-pregnancy (kg)	67.56 ± 14.54
Weight; term (kg)	85.43 ± 14.30
BMI; pre-pregnancy (kg/m^2^)	24.91 ± 4.81
BMI; term (kg/m^2^)	29.62 ± 5.80
Weeks of gestation (*n*)	38.80 ± 1.56
Smoking (*n* (%))	10 (0.14)
Alcohol consumption (*n* (%))	8 (0.11)
Previous live births (*n* (%))	26 (0.37)
Daily Calcium Supplementation (*n* (%))	37 (0.56)
Daily Calcium Supplementation (mg)	423.07 ± 319.07
Daily dietary Calcium intake during 3rd trimester (mg)	792.5 ± 334.0
Daily dietary vitamin D intake during 3rd trimester (mcg)	2.9 ± 1.2
Paternalheight (cm)	177.85 ± 6.14
**Neonatal**	
Number (*n*)	66
Gender; Males (*n*(%))	38 (0.58)
Height (cm)	50.48 ± 1.96
Weight (g)	3292.12 ± 414.25

**Table 2:** **Birth neonatal anthropometry (maternal 25(OH)D ≤ 25 nmol/L)according to maternal VDBP polymorphisms**. If Levene’s Test for Equality of Variances *p* > 0.05 then Equal variances assumed Sig (2-tailed) *p*-values of T-Tests were given. If Levene’s Test for Equality of Variances *p* < 0.05 then Equal variances not assumed Sig (2-tailed) *p*-values of T-Tests were given. * The data and *p*-values adjusted for maternal height (cm), BMI pre-pregnancy (kg/m^2^), BMI terminal (kg/m^2^), UVB exposure, and weeks of gestation by One-way analysis of covariance (ANCOVA). Numbers in italics are after adjustments. ***^¥^ Observed power***.

SNP	Genotype	N	Height (cm)	Weight (g)	HeadCirc/ce (cm)	Neck–rumplength (cm)	Chest Circ/ce (cm)	AbdominalCirc/ce (cm)	AbdominalCirc/Ce iliac (cm)	Skin Fold Abdominal (cm)	Skin fold High Anterior (cm)	High Thigh Circ/ce (cm)	Middle Thigh Circ/ce (cm)	UpperArmLength (cm)	LowerArmRadialCirc/ce (cm)	LowerLegCalfCirc/ce (cm)	FemurLength(cm)	Knee–HeelLength(cm)
**rs2298850**	CC	8	51.8 ± 2.6 (51.1 ± 2.6)	3483.8 ± 509.2 (3436.7 ± 546.0)	36.7 ± 7.4	17.2 ± 2.8	31.3 ± 1.6	28.5 ± 1.7	26.4 ± 1.9	2.9 ± 0.3	3.6 ± 0.5	14.9 ± 0.9	13.3 ± 0.7	13.6 ± 0.8 (13.4 ± 0.5)	8.9 ± 0.6	10.3 ± 0.8	10.0 ± 0.7	9.3 ± 0.7
	CG +GG	10	49.1 ± 1.2 (48.9 ± 1.2)	3067.8 ± 164.9 (3037.5 ± 147)	34.0 ± 1.2	17.3 ± 1.2	29.8 ± 2.0	27.2 ± 1.7	25.1 ± 1.1	2.8 ± 0.3	3.8 ± 0.5	15.1 ± 1.1	13.1 ± 0.8	13.0 ± 0.6 (13.0 ± 0.6)	8.9 ± 0.6	10.3 ± 0.5	9.7 ± 0.6	9.4 ± 0.5
	***p*-value**		***0.02*** (0.19) * ***0.25 ^¥^***	***0.06*** (0.24) * ***0.20 ^¥^***	0.34	0.96	0.10	0.14	0.10	0.36	0.36	0.69	0.57	***0.05*** (0.47) * ***0.10 ^¥^***	0.90	0.96	0.47	0.79
**rs4588**	CC	7	51.5 ± 2.6	3411.4 ± 503.7	37.1 ± 7.9	17.2 ± 3.0	31.1 ± 1.6	28.2 ± 1.7	26.2 ± 1.9	2.9 ± 0.3	3.7 ± 0.5	14.9 ± 1.0	13.2 ± 0.7	13.6 ± 0.8	8.7 ± 0.6	10.2 ± 0.8	9.9 ± 0.7	9.2 ± 0.7
	CA +AA	11	49.6 ± 1.9	3160.0 ± 330.5	34.0 ± 1.2	17.3 ± 1.2	30.1 ± 2.1	27.5 ± 1.9	25.4 ± 1.3	2.8 ± 0.3	3.8 ± 0.5	15.1 ± 1.1	13.1 ± 0.8	13.0 ± 0.6	9.0 ± 0.6	10.0 ± 0.5	9.8 ± 0.6	9.4 ± 0.5
	***p*-value**		0.10	0.23	0.34	0.92	0.28	0.41	0.27	0.50	0.55	0.60	0.86	0.10	0.49	0.86	0.81	0.48
**rs7041**	GG	5	51.5 ± 2.1	3356.0 ± 349.5	38.0 ± 9.5	17.1 ± 3.6	31.3 ± 1.7	27.7 ± 1.7	26.0 ± 1.7	2.8 ± 0.2	3.8 ± 0.6	14.7 ± 0.9	13.1 ± 0.8	13.8 ± 1.0 (13.4 ± 0.6)	8.7 ± 0.6	10.4 ± 0.9	9.8 ± 0.8	9.0 ± 0.8
	GT +TT	13	49.9 ± 2.2	3225.0 ± 448.9	34.1 ± 1.1	17.3 ± 1.2	30.1 ± 2.0	27.8 ± 1.9	25.6 ± 1.6	2.9 ± 0.3	3.7 ± 0.5	15.2 ± 1.0	13.2 ± 0.8	13.1 ± 0.6 (13.1 ± 0.6)	8.9 ± 0.6	10.2 ± 0.6	9.9 ± 0.6	9.5 ± 0.5
	***p*-value**		0.21	0.57	0.41	0.83	0.25	0.95	0.66	0.62	0.93	0.39	0.83	***0.08*** (0.51) * ***0.10 ^¥^***	0.43	0.75	0.81	0.19

**Table 3:** **Birth neonatal anthropometry (maternal 25(OH)D ≤ 50 nmol/L)according to maternal VDBP polymorphisms.** If Levene’s Test for Equality of Variances *p* > 0.05 then Equal variances assumed Sig (2-tailed) *p*-values of T-Tests were given. If Levene’s Test for Equality of Variances *p* < 0.05 then Equal variances not assumed Sig (2-tailed) *p*-values of T-Tests were given. * The data and *p*-values adjusted for maternal height (cm), BMI pre-pregnancy (kg/m^2^), BMI terminal (kg/m^2^), UVB exposure, and weeks of gestation by One-way analysis of covariance (ANCOVA), Numbers in italics are after adjustments. ***^¥^ Observed power***.

SNP	Genotype	N	Height (cm)	Weight (g)	HeadCirc/ce (cm)	Neck–rumplength (cm)	ChestCirc/ce (cm)	AbdominalCirc/ce (cm)	AbdominalCirc/ce iliac (cm)	Skin Fold Abdominal (cm)	Skin Fold High Anterior (cm)	High Thigh Circ/ce (cm)	Middle Thigh Circ/ce (cm)	UpperArmLength (cm)	LowerArmRadialCirc/ce (cm)	LowerLegCalfCirc/ce (cm)	FemurLength(cm)	Knee–HeelLength(cm)
**rs2298850**	CC	17	51.1 ± 2.2 (50.8 ± 2.4)	3468.2 ± 415.6 (3444.5 ± 446.0)	35.2 ± 5.2	17.5 ± 2.3	31.4 ± 2.2 (31.5 ± 2.2)	28.5 ± 2.1	26.3 ± 1.8 (26.3 ± 1.8)	2.9 ± 0.3	3.7 ± 0.5	15.6 ± 1.7	13.6 ± 1.3	13.7 ± 0.6 (13.6 ± 0.5)	9.0 ± 0.7	10.4 ± 0.8	10.0 ± 0.6	9.3 ± 0.5
	CG +GG	26	50.0 ± 1.4 (49.7 ± 1.3)	3169.6 ± 36 (3090 ± 330)	33.9 ± 1.2	17.8 ± 1.3	30.4 ± 1.7 (30.2 ± 1.8)	27.6 ± 1.6	25.4 ± 1.3 (25.3 ± 1.3)	3.0 ± 0.7	3.9 ± 0.5	15.2 ± 1.0	13.2 ± 0.8	13.3 ± 0.6 (13.3 ± 0.6)	8.9 ± 0.6	10.2 ± 0.7	9.9 ± 0.6	9.2 ± 0.5
	***p*-value**		***0.09*** (0.16) * ***0.28 ^¥^***	***0.02*** (0.06) * ***0.48 ^¥^***	0.22	0.56	***0.09*** (0.09) * ***0.40 ^¥^***	0.11	***0.07*** (0.12) * ***0.34 ^¥^***	0.64	0.55	0.41	0.14	***0.04*** (0.39) * ***0.14 ^¥^***	0.77	0.30	0.51	0.35
**rs4588**	CC	16	50.9 ± 2.2	3435.6 ± 406.2 (3444.5 ± 446)	35.3 ± 5.3	17.5 ± 2.4	31.3 ± 2.2	28.4 ± 2.1	26.2 ± 1.8	2.9 ± 0.4	3.4 ± 0.5	15.6 ± 1.8	13.6 ± 1.4	13.7 ± 0.6 (13.6 ± 0.5)	9.0 ± 0.8	10.4 ± 0.8	10.0 ± 0.6	9.3 ± 0.5
	CA +AA	27	50.1 ± 1.6	3202.4 ± 396.4 (3090.0 ± 330)	33.9 ± 1.1	17.8 ± 1.2	30.5 ± 1.7	27.7 ± 1.7	25.5 ± 1.4	3.0 ± 0.7	3.8 ± 0.5	15.2 ± 1.0	13.2 ± 0.8	13.3 ± 0.6 (13.3 ± 0.6)	9.0 ± 0.6	10.2 ± 0.7	9.9 ± 0.6	9.2 ± 0.5
	***p*-value**		0.22	***0.08*** (0.06) * ***0.48 ^¥^***	0.20	0.58	0.16	0.22	0.17	0.63	0.73	0.42	0.18	***0.05*** (0.39) * ***0.14 ^¥^***	0.99	0.37	0.70	0.59
**rs7041**	GG	11	51.4 ± 1.8 (51.4 ± 1.9)	3428.0 ± 368.2	35.8 ± 6.4	17.7 ± 2.8	31.3 ± 2.2	28.3 ± 2.4	26.1 ± 1.9	2.8 ± 0.4	3.8 ± 0.6	15.4 ± 1.7	13.5 ± 1.4	13.7 ± 0.7	9.0 ± 0.9	10.5 ± 0.9	10.0 ± 0.7	9.3 ± 0.6
	GT +TT	32	50.1 ± 1.8 (49.8 ± 1.7)	3250.0 ± 421.0	34.0 ± 1.1	17.6 ± 1.3	30.6 ± 1.8	27.9 ± 1.7	25.7 ± 1.4	3.0 ± 0.6	3.8 ± 0.5	15.3 ± 1.2	13.3 ± 0.9	13.4 ± 0.6	8.9 ± 0.6	10.2 ± 0.7	9.9 ± 0.6	9.2 ± 0.5
	***p*-value**		***0.07*** (0.07) * ***0.45 ^¥^***	0.24	0.36	0.97	0.34	0.54	0.40	0.38	0.94	0.91	0.54	0.12	0.76	0.31	0.65	0.99

**Table 4:** **Birth neonatal anthropometry (maternal 25(OH)D ≤ 75 nmol/L)according to maternal VDBP polymorphisms.** If Levene’s Test for Equality of Variances *p* > 0.05 then Equal variances assumed Sig (2-tailed) *p*-values of T-Tests were given. If Levene’s Test for Equality of Variances *p*< 0.05 then Equal variances not assumed Sig (2-tailed) *p*-values of T-Tests were given. If Levene’s Test for Equality of Variances *p* < 0.05 then Equal variances not assumed Sig (2-tailed) *p*-values of T-Tests were given. * The data and *p*-values adjusted for maternal height (cm), BMI pre-pregnancy (kg/m^2^), BMI terminal (kg/m^2^), UVB exposure, and weeks of gestation by One-way analysis of covariance (ANCOVA). Numbers in italics are after adjustments. ***^¥^ Observed power***.

SNP	Genotype	N	Height (cm)	Weight (g)	HeadCirc/ce (cm)	Neck–rumplength (cm)	ChestCirc/ce (cm)	AbdominalCirc/ce (cm)	Abdominal Circ/ce iliac (cm)	Skin Fold Abdominal (cm)	Skin Fold High Anterior (cm)	High Thigh Circ/ce (cm)	Middle Thigh Circ/ce (cm)	UpperArmLength (cm)	LowerArmRadialCirc/ce (cm)	LowerLegCalfCirc/ce (cm)	FemurLength(cm)	Knee–HeelLength(cm)
**rs2298850**	CC	22	50.6 ± 2.3	3372.7 ± 435.3	35.0 ± 4.7	17.3 ± 2.1	31.0 ± 2.2	28.1 ± 2.2	25.9 ± 1.9	2.9 ± 0.3	3.8 ± 0.5	15.1 ± 1.8	13.3 ± 1.4	13.9 ± 1.3	8.9 ± 0.7	10.2 ± 0.8	10.0 ± 0.6	9.3 ± 0.5
	CG +GG	34	50.3 ± 1.5	3244.2 ± 398.2	34.1 ± 1.2	18.0 ± 1.9	30.8 ± 1.8	28.1 ± 1.9	25.8 ± 1.5	3.0 ± 0.6	3.9 ± 0.4	15.4 ± 1.2	13.3 ± 1.0	13.5 ± 0.8	9.0 ± 0.6	10.3 ± 0.8	9.9 ± 0.6	9.1 ± 0.5
	***p*-value**		0.59	0.27	0.31	0.24	0.67	0.94	0.89	0.52	0.61	0.51	0.95	0.23	0.51	0.74	0.81	0.22
**rs4588**	CC	21	50.4 ± 2.2	3343.3 ± 423.0	35.0 ± 4.8	17.3 ± 2.2	30.9 ± 2.2	27.9 ± 2.2	25.8 ± 1.9	2.9 ± 0.3	3.8 ± 0.5	15.1 ± 1.9	13.3 ± 1.4	13.9 ± 1.3	8.9 ± 0.7	10.2 ± 0.9	9.9 ± 0.6	9.3 ± 0.5
	CA +AA	35	50.4 ± 1.6	3267.5 ± 413.3	34.1 ± 1.1	18.0 ± 1.9	30.8 ± 1.8	28.1 ± 1.9	25.9 ± 1.5	3.0 ± 0.6	3.8 ± 0.4	15.4 ± 1.2	13.3 ± 1.0	13.5 ± 0.8	9.0 ± 0.6	10.3 ± 0.8	9.9 ± 0.6	9.2 ± 0.5
	***p*-value**		0.97	0.52	0.29	0.25	0.88	0.71	0.84	0.51	0.79	0.52	0.82	0.23	0.35	0.65	0.99	0.39
**rs7041**	GG	13	50.8 ± 2.1	3401.7 ± 373.6	35.5 ± 5.9	17.6 ± 2.6	31.1 ± 2.1	28.0 ± 2.4	26.0 ± 1.9	2.8 ± 0.3	3.8 ± 0.6	15.1 ± 1.7	13.3 ± 1.4	14.1 ± 1.5 (14.1 ± 1.9)	9.0 ± 0.8	10.3 ± 0.9	10.0 ± 0.7	9.3 ± 0.6
	GT +TT	42	50.3 ± 1.8	3267.1 ± 425.6	34.1 ± 1.1	17.8 ± 1.9	30.8 ± 1.9	28.1 ± 1.9	25.8 ± 1.6	3.0 ± 0.6	3.8 ± 0.4	15.4 ± 1.4	13.3 ± 1.1	13.5 ± 0.7 (13.5 ± 0.7)	9.0 ± 0.6	10.2 ± 0.8	9.9 ± 0.6	9.2 ± 0.5
	***p*-value**		0.43	0.33	0.43	0.80	0.58	0.84	0.86	0.31	0.99	0.63	0.92	***0.08*** (0.10) * ***0.37 ^¥^***	0.92	0.63	0.63	0.55

**Table 5:** **Birth neonatal anthropometry (neonatal 25(OH)D ≤ 25 nmol/L) according to neonatal VDBP polymorphisms.** If Levene’s Test for Equality of Variances *p* > 0.05 then Equal variances assumed Sig (2-tailed) *p*-values of T-Tests were given. If Levene’s Test for Equality of Variances *p*< 0.05 then Equal variances not assumed Sig (2-tailed) *p*-values of T-Tests were given. * The data and *p*-values adjusted for maternal height (cm), BMI pre-pregnancy (kg/m^2^), BMI terminal (kg/m^2^), UVB exposure, and weeks of gestation by One-way analysis of covariance (ANCOVA) Numbers in italics are after adjustments. ***^¥^ Observed power***.

SNP	Genotype	N	Height (cm)	Weight (g)	HeadCirc/ce (cm)	Neck–rumplength (cm)	ChestCirc/ce (cm)	AbdominalCirc/ce (cm)	Abdominal Circ/ce iliac (cm)	Skin fold abdominal (cm)	Skin Fold High anterior (cm)	High Thigh Circ/ce (cm)	Middle Thigh Circ/ce (cm)	UpperArmLength (cm)	LowerArmRadialCirc/ce (cm)	LowerLegCalfCirc/ce (cm)	FemurLength(cm)	Knee–HeelLength(cm)
**rs2298850**	CC	13	50.7 ± 2.1	3330.8 ± 367.5	35.8 ± 5.8	17.4 ± 2.6	31.3 ± 2.6	28.3 ± 2.5	26.3 ± 2.1	2.9 ± 0.3	3.7 ± 0.6	15.3 ± 1.6	13.5 ± 1.5	13.6 ± 0.7 (13.6 ± 0.5)	9.1 ± 0.8	10.6 ± 0.8 (10.5 ± 0.8)	10.0 ± 0.6	9.6 ± 0.6 (9.4 ± 0.7)
	CG +GG	12	50.3 ± 2.0	3146.4 ± 323.2	33.8 ± 1.2	17.6 ± 1.1	30.3 ± 1.5	27.5 ± 1.4	25.5 ± 0.8	2.9 ± 0.3	4.0 ± 0.4	15.5 ± 0.8	13.3 ± 0.6	13.0 ± 0.5 (13.0 ± 0.5)	8.9 ± 0.3	10.1 ± 0.4 (10.2 ± 0.3)	9.9 ± 0.6	9.1 ± 0.4 (9.1 ± 0.4)
	***p*-value**		0.62	0.20	0.26	0.77	0.23	0.33	0.20	0.65	0.11	0.69	0.60	***0.03*** (0.05) * ***0.51 ^¥^***	0.26	***0.05*** (0.26) * ***0.20 ^¥^***	0.44	***0.04*** (0.38) * ***0.13 ^¥^***
**rs4588**	CC	12	50.4 ± 2.0	3275.8 ± 323.3	35.9 ± 6.1	17.4 ± 2.7	31.2 ± 2.7	28.2 ± 2.5	26.2 ± 2.1	2.9 ± 0.3	3.7 ± 0.5	15.2 ± 1.6	13.5 ± 1.5	13.6 ± 0.7 (13.6 ± 0.5)	9.1 ± 0.8	10.6 ± 0.8 (10.5 ± 0.8)	10.0 ± 0.6	9.5 ± 0.6
	CA +AA	13	50.6 ± 2.2	3216.7 ± 392.8	33.8 ± 1.1	17.6 ± 1.1	30.5 ± 1.6	27.7 ± 1.5	25.7 ± 1.0	2.9 ± 0.3	3.8 ± 0.4	15.4 ± 0.8	13.3 ± 0.6	13.1 ± 0.5 (13.0 ± 0.5)	8.9 ± 0.4	10.2 ± 0.4 (10.2 ± 0.3)	9.9 ± 0.6	9.2 ± 0.4
	***p*-value**		0.85	0.69	0.24	0.79	0.40	0.60	0.44	0.75	0.22	0.71	0.71	***0.06*** (0.05) * ***0.51 ^¥^***	0.47	***0.09*** (0.26) * ***0.20 ^¥^***	0.68	0.10
	GG	7	51.0 ± 2.3	3288.6 ± 315.8	37.2 ± 7.9	17.1 ± 3.5	31.7 ± 3.0	28.2 ± 3.2	26.2 ± 2.4	2.9 ± 0.3	4.0 ± 0.5	15.0 ± 1.8	13.6 ± 1.9	13.8 ± 0.8 (13.6 ± 0.6)	9.3 ± 1.0	10.7 ± 1.1	10.0 ± 0.7	9.6 ± 0.8
	GT +TT	18	50.3 ± 1.9	3228.8 ± 375.1	33.9 ± 1.1	17.6 ± 1.1	30.5 ± 1.7	27.8 ± 1.5	25.8 ± 1.2	2.9 ± 0.3	3.8 ± 0.5	15.5 ± 1.0	13.3 ± 0.7	13.1 ± 0.5 (13.1 ± 0.5)	8.9 ± 0.4	10.3 ± 0.4	9.9 ± 0.6	9.2 ± 0.4
	***p*-value**		0.45	0.72	0.31	0.72	0.36	0.74	0.71	0.64	0.46	0.58	0.77	***0.01*** (0.40) * ***0.13 ^¥^***	0.32	0.40	0.76	0.32

**Table 6:** **Birth neonatal anthropometry (neonatal 25(OH)D ≤ 50 nmol/L) according to neonatal VDBP polymorphisms**. If Levene’s Test for Equality of Variances *p* > 0.05 then Equal variances assumed Sig (2-tailed) *p*-values of T-Tests were given. If Levene’s Test for Equality of Variances *p* < 0.05 then Equal variances not assumed Sig (2-tailed) *p*-values of T-Tests were given. * The data and *p*-values adjusted for maternal height (cm), BMI pre-pregnancy (kg/m^2^), BMI terminal (kg/m^2^), and weeks of gestation by One-way analysis of covariance (ANCOVA) Numbers in italics are after adjustments. ***^¥^ Observed power***.

SNP	Genotype	N	Height (cm)	Weight (g)	HeadCirc/ce (cm)	Neck–rumplength (cm)	ChestCirc/ce (cm)	AbdominalCirc/ce (cm)	AbdominalCirc/ce Iliac (cm)	Skin Fold Abdominal (cm)	Skin Fold High Anterior (cm)	High Thigh Circ/ce (cm)	Middle Thigh Circ/ce (cm)	UpperArmLength (cm)	LowerArmRadialCirc/ce (cm)	LowerLegCalfCirc/ce (cm)	FemurLength(cm)	Knee–HeelLength(cm)
**rs2298850**	CC	29	50.4 ± 2.2	3254.1 ± 440.7	35.0 ± 3.9	17.0 ± 2.6 (16.8 ± 2.8)	30.8 ± 2.3	27.8 ± 2.3	25.8 ± 1.9	2.9 ± 0.3	3.8 ± 0.5	15.0 ± 1.4 (15.0 ± 1.6)	13.2 ± 1.3	13.8 ± 1.1	9.0 ± 0.7	10.2 ± 0.9	10.0 ± 0.6	9.4 ± 0.6 (9.2 ± 0.6)
	CG + GG	33	50.6 ± 1.7	3335.6 ± 355.5	34.1 ± 1.2	18.3 ± 1.5 (18.3 ± 1.7)	31.0 ± 1.7	28.4 ± 1.8	26.1 ± 1.6	3.0 ± 0.7	3.9 ± 0.4	15.8 ± 1.4 (15.8 ± 1.3)	13.5 ± 1.1	13.5 ± 0.8	9.0 ± 0.6	10.3 ± 0.8	10.0 ± 0.6	9.1 ± 0.4 (9.2 ± 0.4)
	***p*-value**		0.66	0.43	0.21	***0.02*** (0.13) * ***0.33 ^¥^***	0.70	0.26	0.50	0.53	0.32	***0.04*** (0.04) * ***0.55 ^¥^***	0.31	0.25	0.94	0.60	0.89	***0.07*** (0.90) * ***0.05 ^¥^***
**rs4588**	CC	27	50.2 ± 2.1	3237.4 ± 386.7	34.9 ± 4.1	17.0 ± 2.7 (16.7 ± 2.9)	30.8 ± 2.3	27.6 ± 2.3	25.6 ± 1.9	2.9 ± 0.3	3.8 ± 0.5	14.9 ± 1.5 (15.0 ± 1.6)	13.1 ± 1.3	13.8 ± 1.2	9.0 ± 0.8	10.2 ± 0.9	10.0 ± 0.4	9.4 ± 0.5 (9.3 ± 0.5)
	CA +AA	35	50.7 ± 1.7	3344.1 ± 404.5	34.1 ± 1.0	18.2 ± 1.5 (18.3 ± 1.6)	31.1 ± 1.7	28.4 ± 1.9	26.2 ± 1.6	3.0 ± 0.6	3.9 ± 0.4	15.8 ± 1.3 (15.8 ± 1.3)	13.6 ± 1.0	13.6 ± 0.8	9.0 ± 0.6	10.4 ± 0.7	9.9 ± 0.7	9.1 ± 0.5 (9.1 ± 0.5)
	***p*-value**		0.40	0.30	0.29	***0.02*** (0.07) * ***0.44 ^¥^***	0.61	0.16	0.24	0.71	0.85	***0.03*** (0.06) * ***0.49 ^¥^***	0.17	0.28	0.62	0.36	0.72	***0.02*** (0.26) * ***0.20 ^¥^***
**rs7041**	GG	16	50.9 ± 2.4	3214.4 ± 458.8	35.5 ± 5.4	16.6 ± 2.9 (16.3 ± 2.8)	30.6 ± 2.5	27.5 ± 2.6	25.5 ± 2.0	3.0 ± 0.3	3.8 ± 0.5	14.6 ± 1.5 (14.5 ± 1.5)	13.0 ± 1.5	13.7 ± 0.7	8.9 ± 0.8	10.1 ± 1.0	9.9 ± 0.5	9.3 ± 0.6
	GT +TT	47	50.3 ± 1.7	3326.2 ± 373.9	34.1 ± 1.1	18.0 ± 1.8 (18.1 ± 2.1)	31.1 ± 1.8	28.3 ± 1.9	26.1 ± 1.6	3.0 ± 0.6	3.8 ± 0.4	15.7 ± 1.4 (15.8 ± 1.3)	13.5 ± 1.0	13.7 ± 1.0	9.0 ± 0.6	10. ± 0.8	10.0 ± 0.6	9.2 ± 0.5
	***p*-value**		0.42	0.34	0.34	**0.02** (0.13) * ***0.33 ^¥^***	0.46	0.21	0.22	0.99	0.88	***0.02*** (0.01) * ***0.74 ^¥^***	0.16	0.99	0.69	0.31	0.80	0.35

**Table 7:** **Birth neonatal anthropometry (neonatal 25 (OH)D ≤75 nmol/L) according to neonatal VDBP polymorphisms**. If Levene’s Test for Equality of Variances *p* > 0.05 then Equal variances assumed Sig (2-tailed) *p*-values of T-Tests were given. If Levene’s Test for Equality of Variances *p* < 0.05 then Equal variances not assumed Sig (2-tailed) *p*-values of T-Tests were given. * The data and *p*-values adjusted for maternal height (cm), BMI pre-pregnancy (kg/m^2^), BMI terminal (kg/m^2^), and weeks of gestation by One-way analysis of covariance (ANCOVA) Numbers in italics are after adjustments. ***^¥^ Observed power***.

SNP	Genotype	N	Height (cm)	Weight (g)	HeadCirc/ce (cm)	Neck–rumplength (cm)	ChestCirc/ce (cm)	AbdominalCirc/ce (cm)	AbdominalCirc/ce Iliac (cm)	Skin fold Abdominal (cm)	Skin Fold High Anterior (cm)	High Thigh Circ/ce (cm)	Middle Thigh Circ/ce (cm)	UpperArmLength (cm)	LowerArmRadialCirc/ce (cm)	LowerLegCalfCirc/ce (cm)	FemurLength(cm)	Knee–HeelLength(cm)
**rs2298850**	CC	29	50.4 ± 2.2	3254.1 ± 440.7	35.0 ± 3.9	17.0 ± 2.6 (16.8 ± 2.8)	30.8 ± 2.3	27.8 ± 2.3	25.8 ± 1.9	2.9 ± 0.3	3.8 ± 0.5	15.0 ± 1.4 (15.0 ± 1.6)	13.2 ± 1.3	13.8 ± 1.1	9.0 ± 0.7	10.2 ± 0.9	10.0 ± 0.6	9.4 ± 0.6 (9.2 ± 0.6)
	CG +GG	33	50.6 ± 1.7	3335.6 ± 355.5	34.1 ± 1.2	18.3 ± 1.5 (18.3 ± 1.7)	31.0 ± 1.7	28.4 ± 1.8	26.1 ± 1.6	3.0 ± 0.7	3.9 ± 0.4	15.8 ± 1.4 (15.8 ± 1.3)	13.5 ± 1.1	13.5 ± 0.8	9.0 ± 0.6	10.3 ± 0.8	10.0 ± 0.6	9.1 ± 0.4 (9.2 ± 0.4)
	***p*-value**		0.66	0.43	0.21	***0.02*** (0.13) * ***0.33 ^¥^***	0.70	0.26	0.50	0.53	0.32	***0.04*** (0.04) * ***0.55 ^¥^***	0.31	0.25	0.94	0.60	0.89	***0.07*** (0.90) * ***0.05 ^¥^***
**rs4588**	CC	27	50.2 ± 2.1	3237.4 ± 386.7	34.9 ± 4.1	17.0 ± 2.7 (16.7 ± 2.9)	30.8 ± 2.3	27.6 ± 2.3	25.6 ± 1.9	2.9 ± 0.3	3.8 ± 0.5	14.9 ± 1.5 (15.0 ± 1.6)	13.1 ± 1.3	13.8 ± 1.2	9.0 ± 0.8	10.2 ± 0.9	10.0 ± 0.4	9.4 ± 0.5 (9.3 ± 0.5)
	CA +AA	35	50.7 ± 1.7	3344.1 ± 404.5	34.1 ± 1.0	18.2 ± 1.5 (18.3 ± 1.6)	31.1 ± 1.7	28.4 ± 1.9	26.2 ± 1.6	3.0 ± 0.6	3.9 ± 0.4	15.8 ± 1.3 (15.8 ± 1.3)	13.6 ± 1.0	13.6 ± 0.8	9.0 ± 0.6	10.4 ± 0.7	9.9 ± 0.7	9.1 ± 0.5 (9.1 ± 0.5)
	***p*-value**		0.40	0.30	0.29	***0.02*** (0.07) * ***0.44 ^¥^***	0.61	0.16	0.24	0.71	0.85	***0.03*** (0.06) * ***0.49 ^¥^***	0.17	0.28	0.62	0.36	0.72	***0.02*** (0.26) * ***0.20 ^¥^***
**rs7041**	GG	16	50.9 ± 2.4	3214.4 ± 458.8	35.5 ± 5.4	16.0 ± 2.9 (16.3 ± 2.8)	30.6 ± 2.5	27.5 ± 2.6	25.5 ± 2.0	3.0 ± 0.3	3.8 ± 0.5	14.6 ± 1.5 (14.5 ± 1.5)	13.0 ± 1.5	13.7 ± 0.7	8.9 ± 0.8	10.1 ± 1.0	9.9 ± 0.5	9.3 ± 0.6
	GT +TT	47	50.3 ± 1.7	3326.2 ± 373.9	34.1 ± 1.1	18.0 ± 1.8 (18.1 ± 2.1)	31.1 ± 1.8	28.3 ± 1.9	26.1 ± 1.6	3.0 ± 0.6	3.8 ± 0.4	15.7 ± 1.4 (15.8 ± 1.3)	13.5 ± 1.0	13.7 ± 1.0	9.0 ± 0.6	10.3 ± 0.8	10.0 ± 0.6	9.2 ± 0.5
	***p*-value**		0.42	0.34	0.34	**0.02** (0.13) * ***0.33 ^¥^***	0.46	0.21	0.22	0.99	0.88	***0.02*** (0.01) * ***0.74 ^¥^***	0.16	0.99	0.69	0.31	0.80	0.35

## Data Availability

The datasets used and/or analyzed during the current study are available from the corresponding author upon reasonable request.
